# Comparing Patterns of Natural Selection across Species Using Selective Signatures 

**DOI:** 10.1371/journal.pgen.0040023

**Published:** 2008-02-08

**Authors:** B. Jesse Shapiro, Eric J Alm

**Affiliations:** 1 Program in Computational and Systems Biology, Massachusetts Institute of Technology, Cambridge, Massachusetts, United States of America; 2 Department of Biological Engineering, Massachusetts Institute of Technology, Cambridge, Massachusetts, United States of America; 3 Department of Civil and Environmental Engineering, Massachusetts Institute of Technology, Cambridge, Massachusetts, United States of America; 4 The Virtual institute of Microbial Stress and Survival, Berkeley, California, United States of America; 5 The Broad Institute of MIT and Harvard, Cambridge, Massachusetts, United States of America; University of Toronto, Canada

## Abstract

Comparing gene expression profiles over many different conditions has led to insights that were not obvious from single experiments. In the same way, comparing patterns of natural selection across a set of ecologically distinct species may extend what can be learned from individual genome-wide surveys. Toward this end, we show how variation in protein evolutionary rates, after correcting for genome-wide effects such as mutation rate and demographic factors, can be used to estimate the level and types of natural selection acting on genes across different species. We identify unusually rapidly and slowly evolving genes, relative to empirically derived genome-wide and gene family-specific background rates for 744 core protein families in 30 γ-proteobacterial species. We describe the pattern of fast or slow evolution across species as the “selective signature” of a gene. Selective signatures represent a profile of selection across species that is predictive of gene function: pairs of genes with correlated selective signatures are more likely to share the same cellular function, and genes in the same pathway can evolve in concert. For example, glycolysis and phenylalanine metabolism genes evolve rapidly in Idiomarina loihiensis, mirroring an ecological shift in carbon source from sugars to amino acids. In a broader context, our results suggest that the genomic landscape is organized into functional modules even at the level of natural selection, and thus it may be easier than expected to understand the complex evolutionary pressures on a cell.

## Author Summary

Natural selection promotes the survival of the fittest individuals within a species. Over many generations, this may result in the maintenance of ancestral traits (conservation through purifying selection), or the emergence of newly beneficial traits (adaptation through positive selection). At the genetic level, long-term purifying or positive selection can cause genes to evolve more slowly, or more rapidly, providing a way to identify these evolutionary forces. While some genes are subject to consistent purifying or positive selection in most species, other genes show unexpected levels of selection in a particular species or group of species—a pattern we refer to as the “selective signature” of the gene. In this work, we demonstrate that these patterns of natural selection can be mined for information about gene function and species ecology. In the future, this method could be applied to any set of related species with fully sequenced genomes to better understand the genetic basis of ecological divergence.

## Introduction

An enormous genetic diversity exists on earth, particularly in the microbial domains of life - yet how much diversity is functional, and what are the important adaptations that serve to partition species into different niches? Adaptive differences can be identified in genes subject to positive Darwinian selection - the evolutionary force that causes advantageous genetic traits to spread in populations, allowing species to differentiate ecologically. Natural selection acts not just on individual proteins, but on the complex assemblage of proteins specified by an organism's genome. Thus, looking for natural selection across the entire genome is valuable for two reasons. First, it allows us to identify systems-level patterns of adaptation - for example, selection on consecutive enzymes in a metabolic pathway. Secondly, it provides a built-in empirical distribution against which outliers (candidates for selection) can be evaluated. In addition, by simultaneously considering multiple genomes, we can compare relative amounts of selection on a gene in different species subject to different ecological constraints.

Much recent work has focused on genome-wide scans for positive selection in human [1,2] and other eukaryotic species (e.g. *Drosophila*, *Plasmodium* [3,4]). Many of these scans rely on skews in polymorphism patterns as selectively favored alleles become fixed in a population [5]. Most such tests for selection assume that neutral polymorphism patterns at each locus are unlinked from the rest of the genome, making selected loci stand out as regions of reduced variation, or unexpectedly long haplotypes [6]. It is thus unclear whether any of these ‘diversity-based' tests (e.g., Tajima's D [7], Fay & Wu's *H* [8]) for positive selection on sexual genomes - which rely on the assumption that recombination occurs between genomic loci - will be amenable to bacteria, in which recombination is decoupled from reproduction, occurring infrequently, and sometimes across species boundaries (horizontal gene transfer; HGT).

Alternative ‘rate-based' approaches to detecting positive selection (in both sexual and asexual species) include finding genes with high rates of amino acid substitution - relative to (i) the rate of evolution in other lineages (relative rates), or (ii) the number of silent substitutions in the gene (nonsynonymous : synonymous substitution ratio; dN/dS) [9]. These approaches may lack power when positive selection affects a small number of sites [6,10], and the latter may be inappropriate as dS becomes saturated with multiple substitutions on long branches. Both approaches may have difficulty distinguishing between positive selection (fixation of beneficial mutations) and relaxed purifying selection (loss of constraint, fixation of neutral or deleterious mutations, for example during population bottlenecks). These two types of selection can, however, be better distinguished by normalizing out demographic effects, and when polymorphism data is available, using independent methods such as the McDonald-Kreitman (MK) test, which compares the ratio of synonymous and nonsynonymous substitution rates within and between groups [11].

In this study, we focus on relative evolutionary rates because our model system, the γ-proteobacteria, span a considerable evolutionary time period over which synonymous substitution rates are saturated in many branches, and because polymorphism data from *Escherichia coli* provide an independent means to estimate the relative contributions of positive selection and relaxed negative selection to elevated evolutionary rates. Nonetheless, we show results from dN/dS profiling for comparison.

The biological factors driving protein evolutionary rates are complex and widely debated [12–16] (for recent reviews see [17,18]). In addition, selection may lead to subtle lineage-specific variation in evolutionary rates. To identify potentially important rate variation from the background of gene family and genome-specific rates, we factor evolutionary rates into three components that contribute to the total evolutionary distance (amino acid substitutions per site) as defined in Equation 1 (where r is the total evolutionary rate, and t is time):

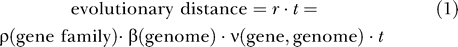
The first and most significant background component (ρ in Equation 1) is related to the protein family: for example, the ribosomal machinery is known to evolve slowly across all sequenced microbes, while surface-exposed proteins often evolve rapidly to avoid predation. The second major contribution (β in Equation 1) is the background rate of evolution that results from the ‘molecular clock' associated with each lineage, perhaps due to between-species differences in population size, generation time, constraint on codon usage, or environmental factors such as UV light exposure [19]. For example, genes from the intracellular parasites of the *Buchnera* genus evolve more rapidly than those in other Enterobacteria. This may be due to frequent population bottlenecks, allowing fixation of neutral or slightly deleterious alleles, or an increased mutation rate [20,21]. Of course, ρ and β are not always independent, and are expected to interact, resulting in evolutionary rate variation that is both gene-specific and species-specific (ν in Equation 1). When a gene evolves at the rate predicted by its gene family and genome, ν will be equal to one. However, when ν deviates from one, this may represent natural selection on different functionality in different genomic/ecological milieus,

Deviations from the ‘expected' rate of protein evolution can be used to detect positive selection and functional diversification between orthologous proteins [22–24], and the ‘expected' background is best estimated empirically, by measuring rates across the entire genome. A recent study demonstrated global differences in evolutionary rate between environments [19], but did not attempt to identify patterns of natural selection on genes in different genomes. The growing number of organisms with fully sequenced genomes provides an opportunity to look for patterns of selection on genomes, and to begin to address a question of fundamental interest: to what extent does differentiation in core, ‘housekeeping' genes drive functional divergence between species across the tree of life? And can we identify genes under selection, and make predictions about their biological/ecological significance?

## Results

**Figure 1:**
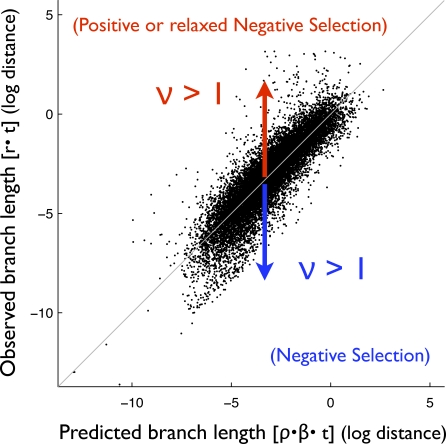
Evolutionary Rate Deviations as Evidence of Natural Selection Observed branch length is plotted against the branch length predicted from gene-specific (ρ) and species-specific (β) effects (see Methods). A total of 16,681 points are plotted, corresponding to 744 orthologous proteins present in 16–30 species. Amino acid substitutions per site are shown on a log2 scale. The gray line corresponds to y = x.

Using a well-sampled sub-tree of γ-proteobacterial genomes, we detected deviations from the ‘expected' rate of evolution (controlling for ρ and β, as described in the Methods and Figure S1), by estimating ν (Equation 1) for each of 744 ‘core' proteins present in single-copy in the majority of species. Of these protein families, 718 (97%) reject a single molecular clock for all species (Likelihood ratio test, *p* < 0.05), indicating substantial species-specific rate variation over the long time scales considered here. As recently shown to be the case among species of fruit flies and fungi [25], protein-family and genome-wide effects account for most (80%) of the variation in evolutionary distances among orthologous proteins in the gammaproteobacteria (Figure 1; Pearson correlation = 0.89, *p* < 2.2e-16); we used the residual variation on each branch as an estimate of ν, and calculated a Z-score (ratio of the mean of ν to its standard deviation over bootstrap-resamplings from the sequence data) to assess confidence in any deviation from ν = 1. As expected, ν correlates well with dN (Pearson's correlation = 0.44, *p* < 2.2e-16), and the correlation is improved substantially once dN is also normalized for protein-family and molecular effects (Pearson's correlation = 0.78, *p* < 2.2e-16). Interestingly, ν correlates less well with normalized dN/dS (Pearson's correlation = 0.11, *p* < 2.2e-16), perhaps due to dS becoming saturated over the long time scales considered, or simply because dN/dS and relative rates (ν) detect different types and magnitudes of selection, thus predicting different sets of selected genes.

**Figure 2:**
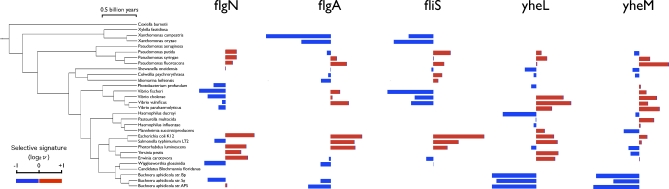
Genes of Common Function Have Similar Selective Signatures Relative rates of evolution are shown for five genes across 30 species. Fast-evolving genes (log2ν > 0) are shown as red bars; slow-evolving genes (log2ν < 0) as blue bars; genes absent in a given species are not shown. The time scale for the phylogeny was estimated using a Bayesian relaxed molecular clock model [52]. Flagellar genes: *flgN* (COG 3418; Flagellar biosynthesis/type III secretory pathway chaperone), *flgA* (COG 1261; Flagellar basal body P-ring biosynthesis protein), *fliS* (COG 1516; Flagellin-specific chaperone). Sulfur metabolism genes: **yheL** (COG 2168; Uncharacterized conserved protein involved in oxidation of intracellular sulfur), *yheM* (COG 2923; Uncharacterized conserved protein involved in oxidation of intracellular sulfur).

When relative rates are overlaid onto the species tree [26], patterns of selection across both genes and species become apparent. For example, genes involved in flagellar biosynthesis (e.g., *flgN, flgA* and *fliS*) are unusually fast-evolving in species of Enterobacteria, while genes putatively involved in sulfur oxidation (*yheL* and *yheM*) are unusually slow-evolving in species of *Buchnera* (Figure 2). As described below, genes involved in the same biological function (e.g., flagellar biosynthesis or sulfur oxidation) tend to have a similar ‘selective signature' (pattern of fast or slow evolution across species). In other words, they evolve in a manner more similar to each other than to genes of a different function. This similarity could be due to genes of the same function being encoded on the same operon (as is the case for *flgA/flgN* and *yheL/yheM*, respectively). Yet *fliS*, which is encoded on a different operon than *flgA/flgN*, has a selective signature similar to as the other flagellar genes (Figure 2), suggesting selection on gene function.

### Selection Acts Coherently at the Level of Function

**Figure 3:**
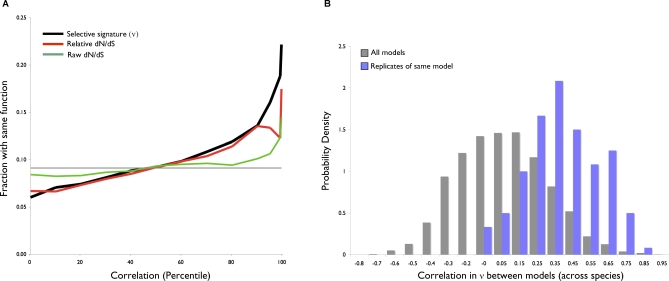
Selection Acts Coherently on Cellular Functions (A) Correlations in ν, dN/dS, and relative dN/dS (normalized as described in Methods) were obtained for the 109,405 gene-pairs with a COG functional category annotation (16 categories, excluding “general” or “unknown” function). Of these pairs, 10,377 have the same COG function, accounting for a proportion of ∼0.09 of the total (plotted as a solid gray line). Pairs were binned according to correlation-percentile in groups of ten percentile points except for the last three (90%–95%, 95%–99%, 99%–100%). Shown is the fraction with common function in each bin. To avoid potential bias, percentiles were calculated separately for genes present in different numbers of species (15 bins ranging from 16 to 30 species). (B) Gene families under the same model of evolution have highly correlated selective signatures. Correlations in ν were obtained for all pairs of simulated gene families, with or without branch variation in dN/dS, and with dN/dS chosen randomly from within ±1 standard deviation of the mean of the observed dN/dS values (range: 0.005–0.26). The distribution of correlations is shown for pairs of gene families with branch variation in dN/dS and that are replicates of the same evolutionary model (light blue). The distribution of all pairwise correlations—including gene families with or without branch variation, and pairs from the same or different models—is also shown (gray).

In addition to the anecdotal cases described above, we examined more generally whether genes of common function tend to experience similar regimes of selection. Indeed, in our overall dataset, pairs of genes sharing the same COG (clusters of orthologous groups [27]) functional annotation have significantly more correlated selective signatures (the vector of ν across all species) than pairs with different functions (Kolmogorov-Smirnov (KS) test, D = 0.12, *p* < 2.2e-16); conversely, genes with similar selective signatures are more likely to share a common function (Figure 3A). This indicates that selection can act coherently at the level of function, and across levels of organization larger than single genes. Considering each functional category in isolation, we find that most functions (11 of 16 COG function categories, excluding ‘general' and ‘unknown' categories) contribute significantly to this effect. Thus, selective signatures are a surprisingly good predictor of common function – a feature that could be useful in the annotation of genes of unknown function, provided that they have orthologs in several species. Correlation in ν is also a significantly better predictor of function than correlation in dN/dS (Figure 3A), or raw evolutionary distance, and the predictive power remains strong even after removing genes used to construct the species tree or genes on the same operon (Figure S3). When dN/dS is normalized by its median for each ortholog and genome to produce a ‘relative' dN/dS measure, it correlates much better with function, almost equal to ν, highlighting the generality of the empirical multi-species approach used in this study.

Our dataset of 744 genes is enriched in highly conserved ‘housekeeping' genes (median dN/dS = 0.047, with 70% of dN/dS values (within 1 standard deviation on a log_2_ scale) ranging from 0.005 to 0.26). Despite this uniformly low range of dN/dS, the subtle rate variation captured by selective signatures is able to identify co-dependencies between genes of related functions. We explicitly tested the ability to detect co-dependencies between genes by simulating codon data for 30 species under 36 different models of evolution, half of which allowed dN/dS to vary on different branches, chosen at random. All models allowed dN/dS to vary among sites. However, for any site, dN/dS was only allowed to range within 1 standard deviation of the mean of the observed data (0.005 to 0.26). For each of the 36 models, 5 replicate datasets were generated, and we treated replicates as genes with known evolutionary co-dependence. We computed ν for each of the resulting 180 simulated genes, and found that in models with branch variation in dN/dS, replicates of the same model had significantly more correlated ν across species than expected (KS test versus all models, D = 0.58, *p* < 2.2e-16; Figure 3B). Thus, when at least some branch variation is present, selective signatures are able to uncover genes with similar evolutionary patterns, even amidst a strong background of purifying selection.

### Patterns of Selection Reflect Ecology

The relationship between selective signatures and gene function is borne out in several genomes in our study. For example, evolution of flagellar proteins appears to be most rapid in some species of Enterobacteria, perhaps reflecting evolutionary ‘arms races' with hosts or predators. In contrast, ion transport/metabolism proteins, especially those involving sulfur, are slowest evolving in *Buchnera aphidicola* APS (Table S3A and S3B), indicating the importance of these proteins in the lifestyle of this intracellular symbiont.

**Figure 4:**
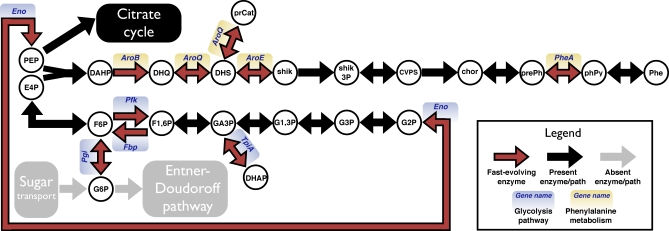
Rapidly Evolving Pathways in *I. loihiensis* Simplified schematic of glycolysis and phenylalanine metabolism in *I. loihiensis*. Metabolic intermediates are denoted by white circles; enzymes by arrows. “Fast-evolving” enzymes, depicted as red arrows, are defined as those with ν in the top 10% of genes in the *I. loihiensis* genome. The names of genes encoding fast-evolving enzymes are shown, highlighted in light blue or orange, respectively for glycolysis or phenylalanine metabolism. Nonfunctional pathways (those with many key enzymes or transporters missing) are shown in gray. Of the “present” enzymes shown in black, only one is slow-evolving (ν < 1) in *Idiomarina:* COG 191, encoding the enzyme fructose bisphosphate aldolase, which interconverts F1,6P and GA3P. Abbreviations for metabolic intermediates: PEP, phoshphenolpyruvate; E4P, erythrose-4-phosphate; DAHP, 7P-2-dehydro-3-deoxy-arabinoheptonate; DHQ, 3-dehydroquinate; DHS, 3-dehydroshikimate; prCat, protocatechuate; shik, shikimate; shik-3P, shikimate-3-phosphate; CVPS, 5-O-(1-carboxyvinyl)-3-phosphoshikimate; chor, chorismate; prePh, prephenate; phPy, phenylpyruvate; Phe, phenylalanine; G6P, glucose-6-phosphate; F6P, fructose-6-phosphate; F1,6P, fructose-1,6-bisphosphate; GA3P, glyceraldehyde-3-phosphate; DHAP, dihydroxyacetone phosphate; G1,3P, glyerate-1,3-bisphospate; G3P, glycerate-3-phosphate; G2P, glycerate-2-phosphate. COG and EC numbers of fast-evolving genes: *AroB:* COG337, EC4.2.3.4; *AroQ:* COG757, EC4.2.1.10; *AroE:* COG169, EC1.1.1.25; *PheA:* COG77, EC4.2.1.51; *Pgi:* COG166, EC5.3.1.9; *Fbp:* COG158, EC3.1.3.11; *Pfk:* COG205, EC2.7.1.11; *TpiA:* COG149, EC5.3.1.1; *Eno:* COG148, EC4.2.1.11.

A deep-sea bacterium that lives at the periphery of hydrothermal vents, *Idiomarina loihiensis*, presents a particularly interesting case study. Having lost many genes essential for sugar metabolism, it relies instead on amino acids as its primary source of energy and carbon [28]. Consistent with disuse of sugar metabolism, we find that glycolysis genes, as well as an upstream phosphotransferase system component (COG2190) have some of the highest values of ν in the *Idiomarina* genome, suggesting relaxed negative selection on this pathway (Figure 4). Moreover, carbohydrate transporters and key glycolytic enzymes in the pentose phosphate and Entner-Doudoroff pathways have been lost in *Idiomarina*, and two of these relatively rapidly evolving enzymes have been lost (COG166 and COG2190) in *Colwellia*, the most closely related sister-taxon of *Idiomarina* in our study. Taken together, these results suggest the relaxation of purifying (negative) selection on this pathway resulting from the disuse of sugars as a carbon source. By contrast, the relatively rapid evolution of amino acid metabolic enzymes in *Idiomarina* might reflect adaptation to growth on amino acids, particularly phenylalanine (Figure 4). Further supporting the idea of a species-specific adaptation in *Idiomarina*, none of the rapidly evolving phenylalanine metabolism genes are also rapidly evolving in *Colwellia*, nor have they been lost in this sister species. The 7 glycolysis genes and 3 phenylalanine biosynthesis genes were also analyzed in PAML [29,30], using models allowing dN/dS to vary among sites and branches, or branches only (Table S4). In the branch-only models, none of these genes had significantly high average dN/dS in *Idiomarina*, but the branch-site models found evidence for a few sites in each gene with unusually high dN/dS in *Idiomarina*. While selective signatures cannot distinguish positive from relaxed negative selection on these genes, the known ecology and genome dynamics suggest positive selection on phenylalanine metabolism and relaxed negative selection on sugar metabolism. Although the true patterns of selection may be more complex, our results paint a broad picture of how the *Idiomarina* core metabolism has been optimized for a diet of amino acids rather than sugars, and lay a path for more targeted follow-up studies.

### Contributions of Purifying and Positive Darwinian Selection

For the cases above, we used biological intuition to discriminate the roles of positive and negative selection on gene evolutionary rates. In general though, natural selection may act to accelerate changes in a protein's sequence (positive selection; ν > 1) or to slow down and constrain its rate of change (negative selection; ν < 1). Alternatively, when negative selection is relaxed, the apparent rate of evolution may increase due to fixation of slightly deleterious mutations (relaxed negative selection; ν > 1). Because these scenarios cannot be distinguished by relative rates methods alone, we employed an independent test for selection (the McDonald-Kreitman (MK) test [11]) using polymorphism data from 473 genes from 24 fully sequenced *E. coli* strains, with *Salmonella enterica* as an outgroup. In the MK test, rather than normalizing according to a sample of distantly related species (as in the selective signatures approach), we normalize according to the expected dN/dS from a within-species polymorphism sample. Specifically, the ratio of synonymous (S) and nonsynonymous (NS) changes at polymorphic sites (within the 24 strains) is compared to the ratio at (nonpolymorphic) divergent sites (comparing *E. coli* to *S. enterica*). The Fixation Index is calculated as FI = (divergent NS/S)/(polymorphic NS/S) [3]. Under neutral evolution, FI is expected to equal 1; under positive selection it may exceed 1, and under negative selection it may be less than 1. We compared the FI values of the 473 genes to their corresponding selective signatures (ν) in *E. coli* and found a significant positive correlation (Pearson's correlation = 0.23, p = 6.5e-7). Although relaxation of negative selection on the branch leading to *S. enterica* could result in high values of FI, at least some of the genes with the highest values of FI are expected to be under positive selection [31]. This demonstrates that relative rate acceleration is often associated with positive selection, and deceleration with purifying selection (for a complete list of selected genes identified by both methods, see Table S5). The correlation between ν and FI is striking because, although the same set of gene families were used to calculate relative rates and the FI, the former used protein sequence while the latter used DNA, and the alignments were performed independently using different sets of species. These results imply that many genes have experienced positive selection since the divergence of *E. coli* and *Salmonella*, despite low overall values of dN/dS.

**Figure 5:**
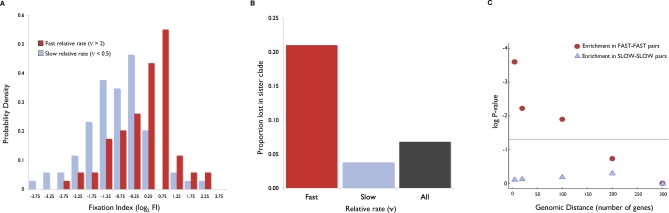
Evidence for Positive and Negative Natural Selection (A) Comparison of relative rates (ν) and Fixation Index. Histograms show the frequency (probability density) distribution of FI values for fast-evolving (ν > 2; dark red; n = 69) and slow-evolving (ν < 0.5; light blue; n = 63) genes. Bins are labelled with the FI value corresponding to their midpoint, on a log2 scale. FI was calculated by counting fixed and polymorphic substitutions at synonymous and nonsynonymous sites, in a sample of 473 COGs (all present in the relatives rates dataset, and consistent with the species tree topology according to the K-H test, as described in the Methods) in 24 *E. coli* strains, using *S. enterica* as an outgroup. (B) Purifying selection and gene deletions. Fast-evolvers (or slow-evolvers) were defined as those genes evolving four times faster (or slower) than expected (ν > 4.0 or ν < 0.25, respectively, for fast and slow, with a Z-score > 1.0). For the fast and slow sets of genes, we counted the number with lost orthologs in the closest sister clade in the species tree. When the sister clade contains multiple species, loss indicates the gene was absent from all species in the clade. Frequency of loss among the fast and slow sets was significantly different than the average over all other genes: higher in the fast-evolving set (Fisher's exact test: Odds Ratio = 3.1, p = 2.4e-7), and lower in the slow-evolving set (Fisher's exact test: Odds Ratio = 0.55, p = 0.01). (C) Evidence for genetic hitchhiking. A binomial test was used to determine whether fast (or slow) evolving genes tend to be clustered in the genome near other fast (or slow) evolving genes across all 30 species combined (ν > 1 or ν < 1, respectively, for fast and slow, with a Z-score > 1.0). Log p-values are plotted for pairs separated by distance-windows of 0–5 genes, 6–20 genes, 21–100 genes, 101–200 genes, and 201–300 genes (points shown indicate the maximum separation). The gray line represents p = 0.05.

When the distributions of FI values are compared between genes with fast (ν > 2) versus slow (ν < 0.5) relative rates (Figure 5A), the difference is very clear. Fast-evolving genes have significantly higher FI values than slow-evolving genes (one-sided KS test; D = 0.43, p = 4.1e-6). The fast and slow subsets are also both significantly different from the mid-range (0.5 < ν < 2) subset of genes (one-sided KS tests: D = 0.17; p = 0.04, and D = 0.30; p = 2.7e-5, respectively for fast and slow). Moreover, the distribution of FI values for fast-evolving genes has a broad shoulder with mean slightly less than 1, and a sharper peak with mean greater than 1 (note the log2 scale in the figure). The simplest interpretation of these results is that increased relative rate reflects both relaxed negative selection and positive selection. Interestingly, the two hypothesized distributions appear to contain a similar number of genes, suggesting that positive selection is about as common as relaxed negative selection as a cause for acceleration of evolutionary rate. This result is largely in agreement with the previous finding that ∼50% of amino acid substitutions between *E. coli* and *S. enterica* were fixed by positive selection [31], with the remaining substitutions due to genetic drift, perhaps resulting from relaxed negative selection or hitchhiking with positively selected mutations (discussed below).

Unusually slowly evolving genes (ν < 0.5), on the other hand, show greater levels of negative selection (low FI) than normal genes (0.5 < ν < 2). While these results may seem unsurprising at first, it is important to note that our evolutionary rates have been normalized for gene family-specific effects, thus even the fastest evolving genes (in terms of ‘raw' rate) will appear ‘slow-evolving' (ν < 1) in about half of the genomes. Conversely, the slowest evolving genes (e.g., the ribosomal machinery) will appear to be ‘fast-evolving' (ν > 1) in about half of the genomes.

To further investigate the role of negative selection, we used gene deletions within a clade as evidence of relaxed negative selection, with the expectation that genes under relaxed selective constraint are lost more frequently. Consistent with a significant role for negative selection in constraining rate variation, genes evolving much more slowly than expected (ν < 0.25) were less likely to have undergone deletion in a sister clade (Figure 5B). Conversely, genes evolving much faster than expected (ν > 4.0) were more likely to have lost their ortholog in a sister clade, pointing toward relaxed negative selection.

### Evidence for Genetic Hitchhiking in Bacteria

In sexually recombining organisms, positively selected mutations are thought to sweep rapidly through the population, lowering effective population size and decreasing the effectiveness of negative selection at linked loci. When sweeps occur faster than recombination can separate the beneficial allele from ‘hitchhikers', clusters of rapidly evolving genes (i.e., one gene under positive selection, and linked genes under relaxed negative selection) can arise [6]. Perhaps unexpectedly for an asexual species, selective sweeps and genetic hitchhiking between linked (∼30 kb apart), but not unlinked loci, have been documented in *E. coli* [32]. Theoretically, there exist regimes of selection and recombination in prokaryotes that would be able to produce a pattern of genetic hitchhiking [33]. Early work on variation across ∼1700 strains of *E. coli* showed genetic linkage between loci separated by ∼45 kb [34] - an estimate largely supported by recent whole-genome scans, which find recombinational segments of up to 100 kb [35]. To determine whether genetic hitchhiking was detectable among fast-evolving genes in this study, we examined proximal pairs of genes (separated on the chromosome by 0–5 genes) and asked whether they showed a tendency to co-evolve - either both ‘fast' (ν > 1), or both ‘slow' (ν < 1). Proximal genes are frequently encoded on the same operon, and are thus expected to be under similar selective pressures due to co-expression and common function. Indeed, we find that pairs of genes predicted to be on the same operon [36] co-evolve in the same direction (either both genes with ν > 1, or both with ν < 1, Z-score > 1; Fisher's Exact Test: Odds Ratio = 3.1, p < 2.2e-16). In fact, selective signature (correlation in ν across species) is a better predictor of operons than dN/dS, and about as accurate as a small compendium of gene expression data from *E. coli* under different experimental conditions (Figure S2). Because these operon effects could confound the detection of hitchhiking, we restricted our analysis to pairs of genes on different operons, transcribed on opposite strands of DNA or separated by at least one gene on the opposite strand. In this operon-free dataset, we observe a slight but statistically significant tendency for fast-evolving genes (ν > 1), but not slow-evolving genes (ν < 1), to cluster together in a genome, not only at distances of 0–5 intervening genes, but even as far as 20–100 genes apart (Figure 5C). Assuming an average gene length of ∼1 kb in prokaryotes [37], clustering of fast-evolving genes up to 100 genes apart (Figure 5C) is very much consistent with earlier predictions [32–35]. Alternatively, genomic mutational hotspots might explain the observed clustering, but this hypothesis is currently difficult to test. Therefore, we tentatively conclude that selective sweeps are occurring in a significant fraction of the 30 species analyzed in this study, and that these sweeps leave a detectable signal in the form of accelerated evolutionary rates.

Taken together, the observed correlations between ν and the Fixation Index (MK test), deletion frequency, and the inferred footprint of genetic ‘hitchhiking' lead us to conclude that ν is reflective of both positive and negative natural selection on core genes.

## Discussion

We have described an approach to detecting selection across genes and genomes. By applying a simple, empirical normalization, we have identified unusually fast- and slow-evolving genes in a phylogeny of 30 bacterial species. Many of these genes are likely targets of natural selection, and are thus among the most important in shaping phenotypic and ecological divergence among species. As genome sequencing continues to outpace detailed phenotypic and functional characterization in many species, efforts to identify the genetic basis underlying ecological differentiation will rely increasingly on sequence-based approaches. Our approach is widely applicable across the tree of life, as it requires only a set of sequenced genomes with common orthologs. Selective signatures have the advantage of detecting subtle gene- and lineage-specific variation in evolutionary rates, but the disadvantage of being limited to core orthologs with representatives in several genomes. For this reason, the timescale and resolution of our approach will depend on the set of species included in the analysis. This study was restricted to extant species (terminal branches), but could easily be extended to include ancestral species (internal branches), providing insight into ancient selective pressures and adaptations.

Relative rates provide information about which genes are evolving unusually rapidly or slowly, but not about what type of natural selection is responsible. We have complemented our between-species relative evolutionary rate estimations with within-species polymorphism data from *E. coli* to show that relative rates are a reasonable and easily estimated predictor of positive and negative selection. In the absence of polymorphism data, relative rates can still yield high-quality predictions of selected genes, which should be followed up by further experimentation to test their functional significance.

### Selective Signatures as a Measure of Natural Selection, or of Niche-Specific *Changes* in Selection

**Figure 6:**
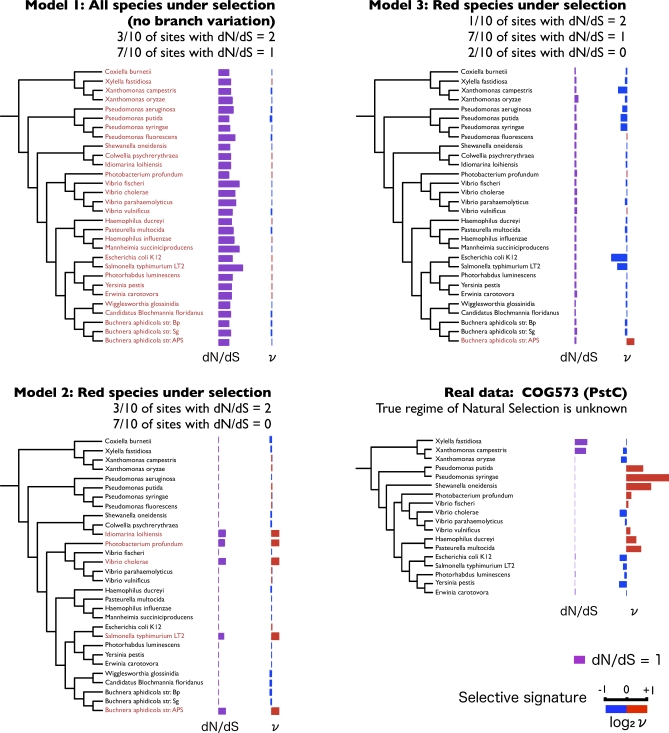
Detection of Positive Selection by dN/dS and ν under Different Evolutionary Models Values of dN/dS and ν (mean over 12 replicates of each model) are shown for three simulation models. Model 1: dN/dS = 2 at 3/10 of sites and dN/dS = 1 at 7/10 of sites, in all species (shown in red). Model 2: dN/dS = 2 at 3/10 of sites and dN/dS = 1 at 7/10 of sites, respectively, for the species shown in red. All other branches had dN/dS = 0 at all sites. Model 3: dN/dS = 2, dN/dS = 1, and dN/dS = 0 at 1/10, 7/10, and 2/10 of sites, respectively, in the species shown in red. All other branches had dN/dS = 1 and dN/dS = 0 at 8/10 and 2/10 of sites, respectively. Values of dN/dS and ν are also shown, as estimated for a real protein family from our dataset of 744 protein families in 30 species.

Even for detecting selection in single genomes, the selective signatures approach can be powerful because it can identify positive (or relaxed negative) selection for genes with low values of dN/dS, while in some other cases selection is more easily detected using dN/dS with a variable branch or branch-site model. To illustrate this, we simulated codon data for 180 genes families under different models of natural selection across our tree of 30 γ-proteobacteria, and calculated dN/dS and ν in each branch (Methods). In cases with elevated dN/dS in all branches (Model 1 in Figure 6), PAML is able to correctly identify all branches under selection. Because there is very little variation among branches, ν is uninformative, despite positive selection in all lineages. When branch variation is present, and selection is strong in some branches but not others (Model 2 in Figure 6), both ν and dN/dS are able to correctly identify the species under selection. Yet when branch variation is present but the branch under selection is only weakly selected (few sites and dN/dS only slightly higher than background), it is identified correctly by ν but not dN/dS (Model 3 in Figure 6). Therefore, ν is well-suited to detect subtle cases of species-specific selection, but is powerless to detect uniform positive selection in all species. This is further demonstrated in an example from a gene family in our dataset: PstC (COG573), which encodes a permease involved in phosphate transport. This gene is highly conserved across 18 species, with dN/dS near zero in most species except *Xylella fastidiosa* and *Xanthomonas campestris*, which have among the highest genome-wide average dN/dS, suggesting the high dN/dS of PstC may be due in part to demographic effects. Despite the lack of information from dN/dS, this gene shows substantial variation in ν across species (Figure 6), which may be related to species-specific ecological factors.

**Figure 7:**
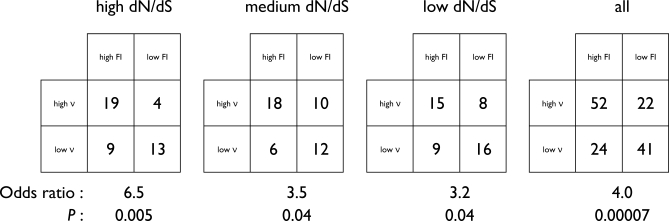
Positive Association of Selective Signatures (ν) and Fixation Index, Independent of dN/dS We counted *E. coli* genes with FI > 1.2 or FI < 0.6 as “high” and “low,” and with log2 ν > 0.5 (ν > 1.4) or log2 ν < −0.5 (ν < 0.7) as “high” and “low.” The genes were divided into sets with relatively high dN/dS ( >0.06), medium (0.02 < dN/dS < 0.06), or low dN/dS (<0.02). Within each set, counts were binned in 2 × 2 contingency tables to calculate the Odds Ratio statistic, with Odds Ratio > 1 indicating positive association between ν and FI. One-sided p-values of Fisher's exact test are shown.

Like the Fixation Index computed in the MK test, selective signatures measure selection relative to a baseline. While the MK identifies selection relative to a baseline of within-population polymorphism, selective signatures test for selection relative to a baseline established by related species. Despite their contrasting and independent data and analytical methods, the two measures tend to overlap significantly in their predictions of natural selection. Moreover, the positive association between them (Figure 7; Odds ratio > 1) persists at high, intermediate, and low levels of dN/dS. The association may be slightly stronger when dN/dS is very high, due to correct identification of strong positive selection by all three methods. Yet even when absolute dN/dS is low, the FI and ν often agree that evolutionary rate is relatively fast, suggesting positive or relaxed negative selection (or strong negative selection, when both FI and ν are low), perhaps on just a few sites. While the MK test may wrongly predict selection after a population bottleneck, leading to between-species fixation of slightly deleterious mutations [10], selective signatures explicitly normalize out such genome-wide effects. On the other hand, if demographic effects are not significant, the MK test has the advantage of distinguishing positive selection from relaxed negative selection, which is not possible with selective signatures. In addition, HGT (e.g., from *S. enterica* to *E. coli*) is expected to reduce the observed divergence, lowering ν without affecting FI or dN/dS. Thus, the intersection of genes predicted by both high FI and ν (see Table S5) provides a more robust prediction of positive selection.

Because selective signatures are also lineage-specific, they represent a measure of niche-specific changes in selection, and have the advantage of being sensitive to substitutions in just a few amino acid sites, provided these are unexpected relative to the gene-family and genome-specific background rates. For example, we identified several *Idiomarina* genes with high values of ν, which corresponded to only a few sites with high dN/dS, while average dN/dS across each gene was low (Table S4). Even if rate acceleration is due to relaxed negative selection rather than positive selection, the change in selection detected by ν is both gene- and lineage-specific, and thus may be relevant to ecological differentiation among species. Genes with similar values of ν in the same species may be part of a co-evolving functional module, and correlations in ν are able to identify such sets of genes (Figures 3, 4, and S2).

### Genome Evolution through Horizontal Transfer and Changes in Core Genes

Can horizontal transfer alter effective protein evolutionary rates, thereby affecting selective signatures? HGT is prevalent in prokaryotes [38,39], especially among closely related taxa [40]. For example, we suspect that homologous recombination (or HGT between close relatives) within ‘species' contributes to the observed clustering of rapidly evolving genes (Figure 5C). HGT can also complicate inferred evolutionary rates in two qualitatively different ways: (i) transfer from distant lineages (or replacement with paralogs) can make distances to sister taxa appear long (and disrupt tree topology); and (ii) transfer between sister taxa does not affect tree topology, but can shorten observed distances. Thus, some of our observed rate variation is likely due to lateral gene flow. We investigated the extent to which HGT affects our results by repeating our analyses with a set of genes more likely to include horizontal gene flow, and concluded that our main findings are not easily attributable to artifacts of HGT (Figures S4–S6). Moreover, our main findings are supported by methods not directly biased by HGT (MK and dN/dS tests).

### Summary

Species are believed to diverge only when they gain the ability to exploit a new ecological niche [41], and this may come about through mutations in existing (core) genes, or acquisition of new genes. It is gaining widespread acceptance that the latter is responsible for many, if not most adaptations [39,42], and possibly ensuing speciation events. Yet, as we demonstrate, core genes are also subject to selection, and likely contribute to differentiation between species over long time spans. Much of this selection is positive, leading to novel adaptations in core genes. Thus, core genes, which are by definition retained in genomes over long periods of time, may be quite dynamic in terms of their precise molecular functionality. The coherence of selective patterns across genes of similar function (those with the same operon, functional annotation, or in the same pathway) is exciting because it suggests that the genomic landscape is organized into functional modules even at the level of natural selection. Thus, it may be easier than anticipated to understand the complex evolutionary pressures acting on genomes. Correlations in selective signatures could be used to identify fitness co-dependencies among genes in much the same way that correlated mRNA expression profiles are used to identify genes connected in the physical or regulatory networks of the cell.

## Materials and Methods

### Estimation of relative evolutionary rates (ν).

To calculate relative evolutionary rates (ν), normalized to remove protein-specific ‘scaffold' constraints (ρ) and species-specific ‘molecular clock' (β) effects, we first constructed a ‘species tree' for 30 species of γ-proteobacteria (see Table S2 for species names and taxonomy IDs). Our tree is based on a concatenation of amino acid sequences for 80 housekeeping genes that occur in single-copy in each genome (Table S1), and have previously been shown to be orthologous and consistent with a single organismal phylogeny [43]. Gene trees were then constructed for 977 putative ‘core' gene families (members of the same cluster of orthologous genes [27], retrieved from the MicrobesOnline database [44]), each occurring as a single copy in at least 16 of the 30 genomes. Multiple sequence alignments (MSAs) were performed using MUSCLE [45], and all gaps were removed, along with one flanking residue on either side. Gene trees were constructed from the resulting MSAs using Tree-Puzzle [46] with a JTT amino acid substitution model [47] and 8 γ-distributed rate categories. Estimation of ν proved to be independent of the substitution model used (see Figure S7 for comparison with WAG model [48]). Gene trees were screened to remove genes that may have resulted from horizontal transfer by excluding all gene families with topologies that conflicted with the species tree topology according to a Kishino-Hasegawa (K-H) test [49] (*p* < 0.05). Of the remaining 744 ‘core' gene families, 99% of the top BLAST hits were to a member of the same Genus, or to a neighboring branch on the species tree. For the 744 gene families consistent with the species tree phylogeny, trees were re-built using the consensus ‘species tree' topology, but with branch lengths estimated separately for each gene. These gene trees were first normalized to remove gene family-specific contributions (ρ) by re-scaling each tree such that the sum of all branch lengths in the tree matched that expected by the species tree (considering only those branches of the species tree that are present in the gene tree). Gene trees were further normalized to remove ‘molecular clock'-type effects (β · *t*) by dividing each branch by the corresponding branch length in the species tree (Figure S1). Only terminal branches (those leading directly to extant species) were used in this study, and branches with near-zero sequence changes were excluded from the analysis. Finally, the resulting relative rates were median centered within each genome, leaving an estimate of ν in which values greater than 1.0 indicate faster than expected evolution (e.g., due to positive or relaxed negative selection), and values smaller than 1.0 indicate slower than expected evolution (e.g., due to increased negative selection). To estimate the significance of the deviation from 1.0 (no unusual selective pressures), we computed 100 replicates of our estimate for ν by nonparametric sequence bootstrapping, and computed a ‘Z-score' as the ratio of the observed log_2_(ν) to the square root of its variance over the bootstrap replicates.

### Estimation of synonymous and nonsynonymous substitution rates (dS and dN).

We used the *codeml* program from the PAML 4.0 package [29] to estimate dN and dS, allowing their ratio to vary freely along branches of the species tree (‘free-ratio' model). Estimates of dN, dS and dN/dS were made for each of the 744 core orthologs described above. To generate ‘relative' values of dN, dS and dN/dS, each of these values was first normalized by its median value for each genome, then by the median for each ortholog. Note the order of normalization steps is reversed from that for relative rates, because there is no prior expectation that dN/dS values across the tree are proportional to evolutionary time/distance.

### Simulation of genes under different models of selection.

We used the *evolver* program from the PAML 4.0 package [29] to simulate gene families of 300 codons in 30 species, using the γ-proteobacteria species tree topology. In the first set of simulations (Figure 3B), we used two classes of sites (occurring at frequency 0.1 and 0.9, respectively), each with a different value of dN/dS, randomly chosen from within ±1 standard deviation of the mean of the observed distribution of dN/dS in our dataset of 744 genes across 30 species. In 18 of the models, dN/dS was not allowed to vary among branches; in the remaining 18 a different dN/dS value was chosen at random for each site class and each branch. For each model, we generated 5 replicate codon sequences in 5 independent runs of *evolver*. In the second set of simulations (Figure 6), we used either 2 or 3 classes of sites (with frequency chosen within the range of 0.1 to 0.9), each with dN/dS of either 2.0, 1.5, 1.1, 1.0, 0.5 or 0. We generated 180 different models, 45 of which did not allow branch variation, and the remaining 135 with 1 to 5 branches under selection, with one site class having a higher dN/dS than the other branches. We generated 12 replicate sequences for each model. For both sets of simulations, we translated the codons to amino acid sequence in order to calculate ν, treating each replicate of each model as a protein family. We also estimated dN/dS in each branch using the free-ratio model in PAML.

### McDonald-Kreitman tests.

Gene families were retrieved from 24 strains of E. coli (including strains of *Shigella*; see Table S2B), and an outgroup, S. enterica. Each gene had exactly one representative in each strain. Genes were assigned to orthologous families using OrthoMCL [50]. Only the 473 gene families corresponding to COGs in the relative rates dataset, and not violating the K-H test, were retained for analysis. We tried excluding genes with a large number of divergent sites relative to polymorphic sites, which might reflect HGT from closely related species, but this did not significantly affect results. Nucleotide sequences were aligned and trimmed using MUSCLE, as described above. Polymorphic substitutions (within the 24 strains of E. coli) and divergent substitutions (fixed between E. coli and *Salmonella*) were counted, and assigned to synonymous or nonsynonymous categories, as previously described [11]. Only codons for which there were no more than two states were retained for analysis, and we always chose the pathway between codons that minimized the number of nonsynonymous changes. An Odds Ratio statistic, the Fixation Index (FI), was then calculated as described in the main text.

## Supporting Information

Figure S1 Example of Tree Normalization and Calculation of ν The normalization procedure is illustrated for two example protein families (columns). We begin with a gene tree of three species ((A,B),C), with branch length ( (substitutions/site) × 10−2) equal to total evolutionary distance (top row). The gene tree is first normalized so that terminal branches all sum to 1. The resulting gene tree, normalized to remove gene-family effects (ρ) is shown in the second row. Each branch in the normalized gene-tree is then divided by the corresponding branch in the normalized species-tree (β, shown in the third row) to yield an estimate of ν for each branch, shown in the bottom row. Values of ν > 1 reflect faster-than-expected evolution; ν < 1 reflect slower-than-expected evolution.
(472 KB TIF)

Figure S2 Operon Prediction by Correlation in ν, dN, dS, and dN/dS Receiver Operating Characteristic curve of several methods for operon prediction. Pairs of genes predicted to be on the same operon are considered “true positives”; pairs on different operons as “false positives.” Correlations were computed between the 157,612 pairs of genes for which both gene expression (from E. coli microarrays under 14–17 different experimental conditions) and relative rate (ν) data was available, and for which the pair is present in at least 16 of 30 species. Of these pairs, 898 are predicted to fall on the same operon in E. coli [36]. To avoid systematic biases in correlations (pairs present in fewer species might achieve higher correlations by chance), the correlations were percentile-ranked together with other genes present in the same number of species. For each level of percent-ranked correlation (in either ν, expression level or raw/normalized dN, dS, or dN/dS, estimated as described in Methods), the percentage of pairs above this level in the different-operon set is plotted against the percentage in the same-operon set. The solid gray line represents random prediction (*y* = *x*). We also assessed the ability of similarity in Fixation Index (FI) to predict E. coli genes on the same operon. For each pair of COGs present in E. coli, we calculated “delta FI” as the absolute difference between the FI of each COG. Delta-FI was percentile-ranked and plotted on the operon-predicting ROC curve. Similarity in FI is a rather poor predictor of operons, perhaps because it a scalar value from one species, rather than a correlation across many species. The E. coli gene expression data was obtained from http://www.microbesonline.org. Correlations in expression level (mean log-ratio of experimental condition to control) were taken between genes for which expression had been measured in at least 14 of 17 experimental conditions, including heat shock (experiment ID 12 [51]), low pH (experiment ID 40; Blattner lab), UV exposure (experiment ID 46, seven time points; GEO (http://www.ncbi.nlm.nih.gov/projects/geo/) accession GSE9), and tryptophan exposure/starvation (experiment ID 47, eight time points; GEO accession GSE7).
(344 KB PDF)

Figure S3 Effect of Normalization Procedure (A) and COGs Used in Species-Tree Construction (B) on Rate-Function Correlation Methods are as described in Figure 3 of the main text.
(A) Comparing normalized rates (ν) to nonnormalized “raw” evolutionary distance. Red lines, correlation in ν between gene-pairs; black lines, correlation in raw evolutionary distance (nonnormalized gene-tree branch lengths) between gene-pairs; filled circles, including all gene pairs; open circles, excluding pairs on the same operon. The distribution of ν-correlations among gene-pairs of common function is significantly more biased toward high correlation than the distribution of raw-distance correlations between gene-pairs of common function (KS test, D = 0.12 and D = 0.10, respectively, for total and same-operon excluded datasets, both p < 2.2e-16), indicating that ν-correlation is a significantly better predictor of function than raw distance, even when same-operon pairs are not considered. The gray line denotes the mean fraction with shared function, over all genes.
(B) Comparing the full set of normalized rates (shown in red) with the set excluding the 80 COGs used to construct the species tree (shown in blue). The mean fraction of genes with shared function for each of these datasets is shown in red or blue, respectively. The distribution of ν-correlations among gene-pairs of common function does not differ significantly between these datasets (KS test, D = 0.02, p > 0.05).
(1.0 MB TIF)

Figure S4 Effect of Topology Violation (Putative HGT) on Rate-Function Correlation ν-Correlations between genes were obtained for gene-pairs falling into three categories: (1) Overall: those among the 744 used throughout this work (red); (2) non-HGT: those among the 161 high-quality orthologs of Lerat et al. [43] (blue); or (3) HGT: those among the 173 orthologs found to significantly violate the species tree topology by the K-H test (green). Only gene-pairs with a COG functional category annotation (16 categories, excluding “general” or “unknown” function), and which occur in at least 16 of 30 species, were used.
(A) All pairs, including genes on the same or different operons; (B) excluding gene-pairs on the same operon. For each case, we binned the pairs according to ν-correlation-percentile (as described in Figure 3) and plotted the fraction with common function in each bin. ν-Correlations are expressed as percentiles to control for possible bias in computing correlation for different vector lengths (ranging from 16–30 species). Solid horizontal lines represent the mean fraction of gene-pairs with shared function, averaged over all values of ν-correlation. For both (A) and (B), the distribution of ν-correlations differed between same-function and different-function pairs only for the Overall and non-HGT sets, but not the HGT set (KS tests: p < 2.2e-16 and NS, respectively, for Overall/non-HGT and HGT set). The distribution of Overall ν-correlations among gene-pairs of common function is significantly different from the HGT distribution for gene-pairs of common function (KS test, D > 0.12, p < 1e-4, for both operon-excluded or total dataset).
(954 KB TIF)

Figure S5 Frequency of Lost Orthologs in Sister Taxa for HGT and Non-HGT Datasets Within each of three datasets (topology violators [failed K-H test, putative HGT], high-confidence [putative non-HGT from Lerat et al. [43]], or Overall [744 genes, passed K-H test]), fast-evolvers (or slow-evolvers) were defined as those genes evolving four times faster (or slower) than expected (ν > 4.0 or ν < 0.25, respectively, for fast and slow, with a Z-score > 1.0). For the fast and slow sets of genes, we counted the number with lost orthologs in the closest sister clade in the species tree. When the sister clade contained multiple species, the loss was only counted if all species in the clade had lost the ortholog. Frequency of loss among the fast (red) and slow (blue) sets was compared to the frequency of loss over all genes (gray) using Fisher's exact test; asterices denote significant differences (p < 0.01). In each of the three datasets, the proportion of lost orthologs was significantly different between fast-evolving and slow-evolving genes. The proportion of fast-evolving genes with lost orthologs did not differ significantly between the HGT and Overall set, nor between the high-confidence non-HGT and Overall set. However, the proportion of slow-evolving genes with lost orthologs was significantly higher in the HGT set than the Overall set (Fisher's exact test, Odds Ratio = 3.2, p = 0.009), but not between the non-HGT and Overall set.
(1.9 MB TIF)

Figure S6 Clustering of “Fast” Genes in HGT and Non-HGT Datasets (A) High confidence dataset (161 probable non-HGT genes [43]).
(B) Topology-violating dataset (173 probable HGT genes; failed K-H test). For each dataset, a binomial test was used to determine whether fast (or slow) evolving genes tend to be clustered near other fast (or slow) evolving genes on the chromosome across all 30 species (ν > 1 or ν < 1, respectively, for fast and slow, with a Z-score > 1.0). The log p-values from the binomial test are plotted against genomic distance between genes. All pairs were on different operons. The p-values are plotted for pairs separated by distance-windows of 0–5 genes, 6–20 genes, 20–100 genes, 100–200 genes, and 200–300 genes. Points shown indicate the maximum separation in each nonoverlapping window. The gray line represents a p-value of 0.05.
(1.1 MB TIF)

Figure S7 Agreement of Values of ν Estimated Using JTT and WAG Substitution Models For each of 744 genes across 30 species, the value of ν estimated under a JTT substitution model is plotted against the value estimated under a WAG substitution model. The species tree topology did not differ significantly between JTT and WAG models implemented in Tree-Puzzle (K-H and S-H tests, p > 0.05); therefore, the same topology was used for both models, but with branch lengths estimated separately to compute ν under each model. The models produce near-identical values of ν (Pearson's correlation = 0.987, R^2^ = 0.974, p < 2.2e-16, n = 16,681).
(114 KB PDF)

Table S1 List of Genes Used To Construct the Species Tree (75 KB PDF)

Table S2 Taxonomy IDs of Species Used in This Study (47 KB PDF)

Table S3 Enrichment of COG Functional Categories in Top 10% Fast- or Slow-Evolving Sets of Genes (46 KB PDF)

Table S4 Evidence for Site-specific Changes in dN/dS in *Idiomarina* Genes (100 KB PDF)

Table S5 Genes Predicted under Selection in E. coli by Selective Signatures and the MK Test (122 KB PDF)
