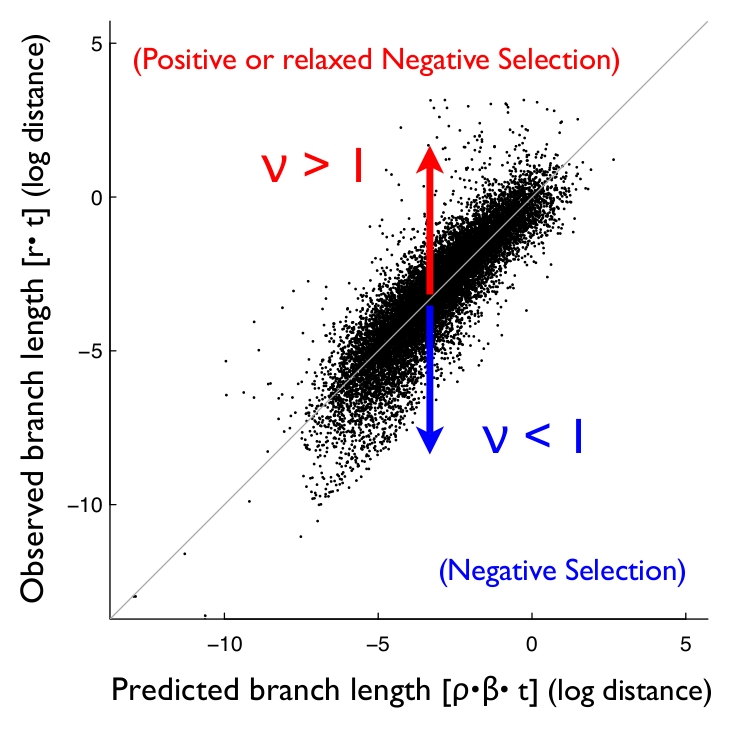# Correction: Comparing Patterns of Natural Selection across Species Using Selective Signatures 

**DOI:** 10.1371/annotation/fced1166-1825-485c-88b9-d6ec950326c8

**Published:** 2008-06-05

**Authors:** B. Jesse Shapiro, Eric J Alm

Figure 1 was displayed incorrectly. The correct Figure 1 is available here: 

**Figure:**