# Supplementary material for: Comparing Patterns of Natural Selection across Species Using Selective Signatures
Source: PLoS Genet. 2008 Feb 8;4(2):e23. doi: 10.1371/journal.pgen.0040023 (PMC2233676; doi:10.1371/journal.pgen.0040023)
Supplement: Supplementary file 1 [file pgen.0040023.sg002.pdf]

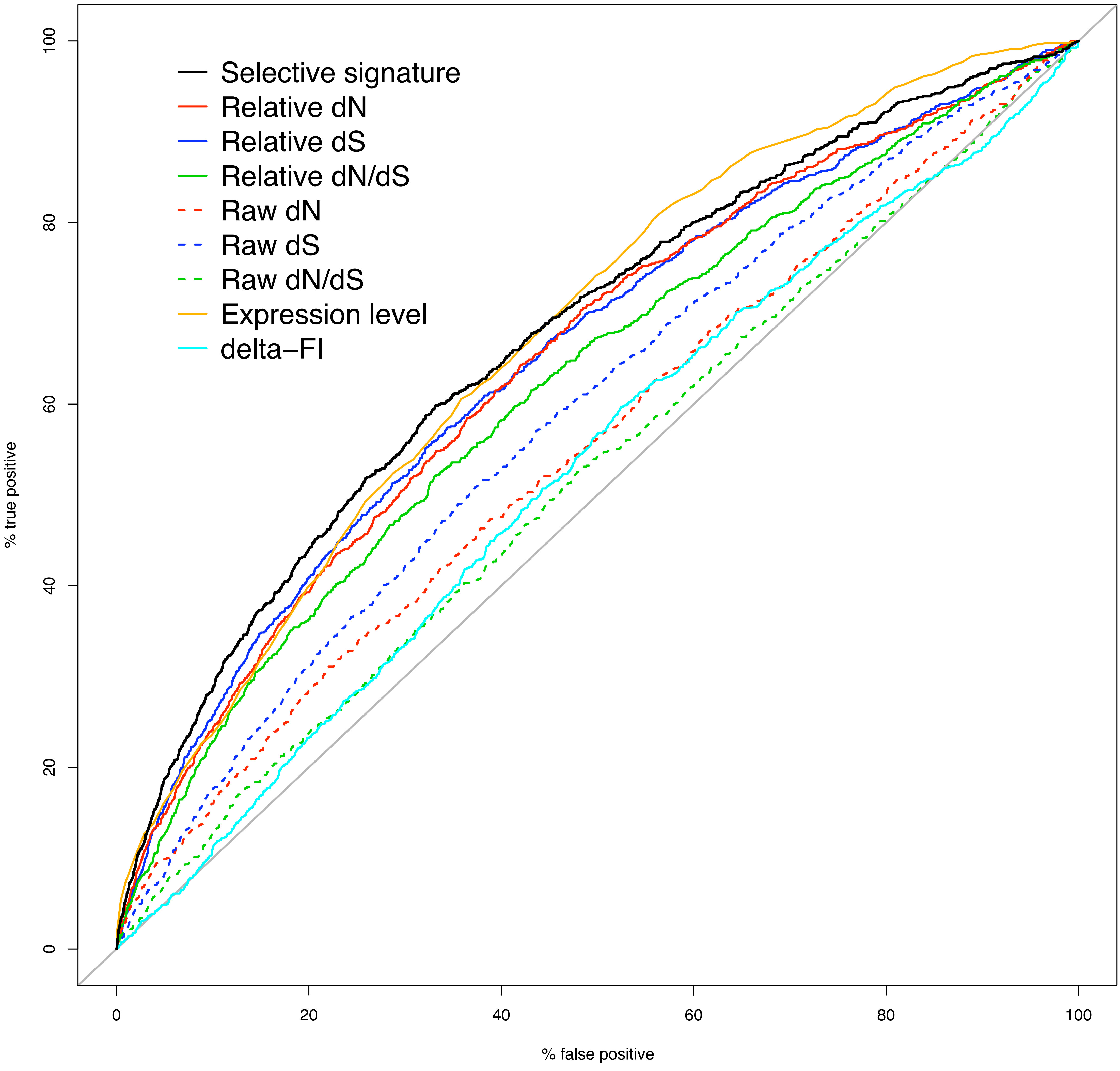

Selective signature
Relative dN
Relative dS
Relative dN/dS
Raw dN
Raw dS
Raw dN/dS
Expression level
delta−FI
% true positive
% false positive
0
20
40
60
80
100
