# Supplementary material for: Comparing Patterns of Natural Selection across Species Using Selective Signatures
Source: PLoS Genet. 2008 Feb 8;4(2):e23. doi: 10.1371/journal.pgen.0040023 (PMC2233676; doi:10.1371/journal.pgen.0040023)
Supplement: Supplementary file 2 [file pgen.0040023.sg007.pdf]

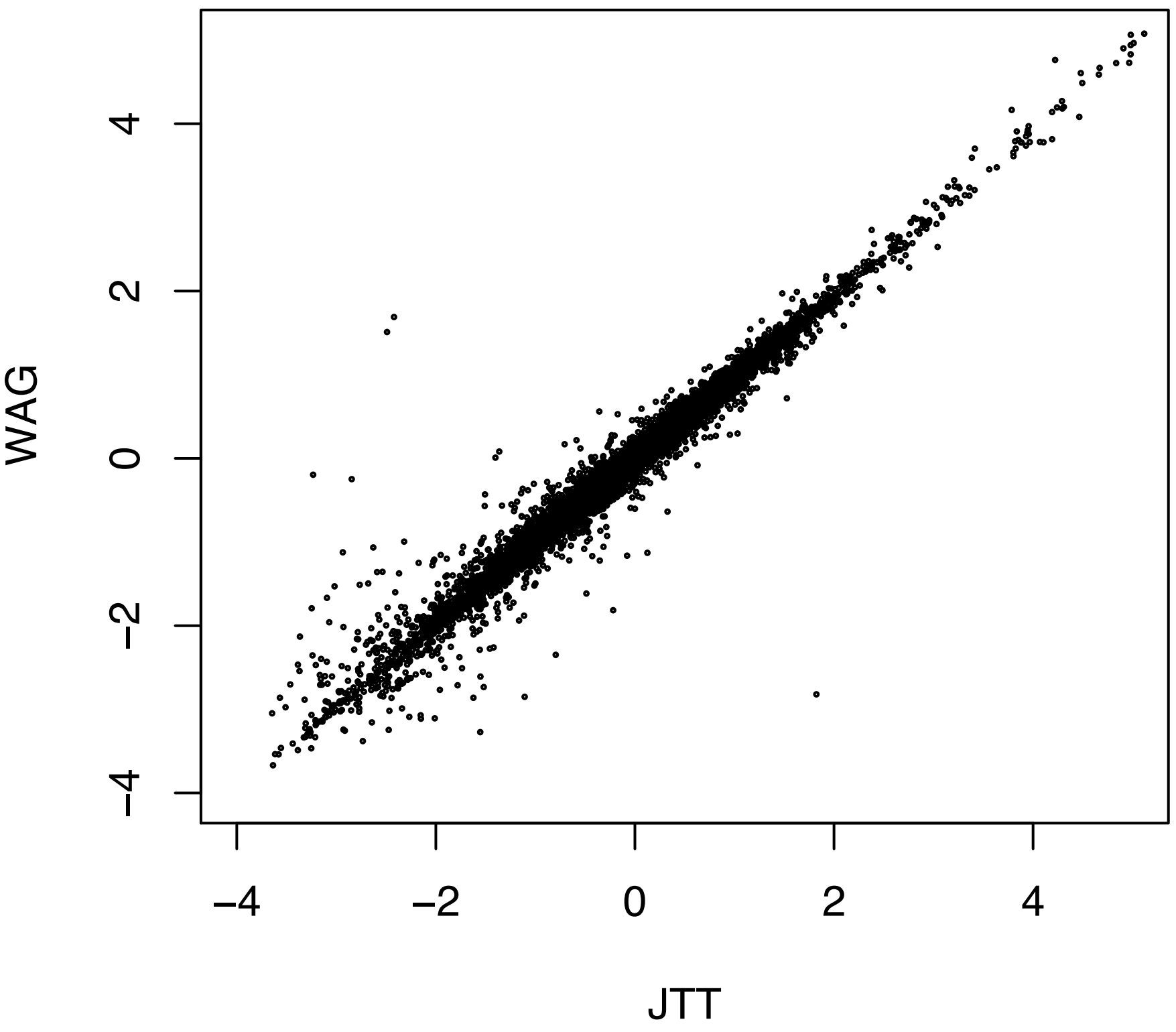
WAG
JTT
−4
−2
0
2
4
−4
−2
0
2
4
