# Supplementary material for: Comparing Patterns of Natural Selection across Species Using Selective Signatures
Source: PLoS Genet. 2008 Feb 8;4(2):e23. doi: 10.1371/journal.pgen.0040023 (PMC2233676; doi:10.1371/journal.pgen.0040023)
Supplement: Supplementary file 3 [file pgen.0040023.st001.pdf]

Supplementary Table 1. List of genes used to construct the species tree.

COG	Name	Functional Category	Description
20	UppS	I	Undecaprenyl pyrophosphate synthase [Lipid metabolism]
30	KsgA	J	Dimethyladenosine transferase (rRNA methylation) [Translation, ribosomal structure and biogenesis]
48	RpsL	J	Ribosomal protein S12 [Translation, ribosomal structure and biogenesis]
51	RpsJ	J	Ribosomal protein S10 [Translation, ribosomal structure and biogenesis]
52	RpsB	J	Ribosomal protein S2 [Translation, ribosomal structure and biogenesis]
60	IleS	J	Isoleucyl-tRNA synthetase [Translation, ribosomal structure and biogenesis]
80	RplK	J	Ribosomal protein L11 [Translation, ribosomal structure and biogenesis]
86	RpoC	K	DNA-directed RNA polymerase, beta' subunit/160 kD subunit [Transcription]
87	RplC	J	Ribosomal protein L3 [Translation, ribosomal structure and biogenesis]
88	RplD	J	Ribosomal protein L4 [Translation, ribosomal structure and biogenesis]
89	RplW	J	Ribosomal protein L23 [Translation, ribosomal structure and biogenesis]
90	RplB	J	Ribosomal protein L2 [Translation, ribosomal structure and biogenesis]
92	RpsC	J	Ribosomal protein S3 [Translation, ribosomal structure and biogenesis]
97	RplF	J	Ribosomal protein L6P/L9E [Translation, ribosomal structure and biogenesis]
98	RpsE	J	Ribosomal protein S5 [Translation, ribosomal structure and biogenesis]
102	RplM	J	Ribosomal protein L13 [Translation, ribosomal structure and biogenesis]
103	RpsI	J	Ribosomal protein S9 [Translation, ribosomal structure and biogenesis]
124	HisS	J	Histidyl-tRNA synthetase [Translation, ribosomal structure and biogenesis]
172	SerS	J	Seryl-tRNA synthetase [Translation, ribosomal structure and biogenesis]
177	Nth	L	Predicted EndoIII-related endonuclease [DNA replication, recombination, and repair]
193	Pth	J	Peptidyl-tRNA hydrolase [Translation, ribosomal structure and biogenesis]
194	Gmk	F	Guanylate kinase [Nucleotide transport and metabolism]
195	NusA	K	Transcription elongation factor [Transcription]
197	RplP	J	Ribosomal protein L16/L10E [Translation, ribosomal structure and biogenesis]
198	RplX	J	Ribosomal protein L24 [Translation, ribosomal structure and biogenesis]
199	RpsN	J	Ribosomal protein S14 [Translation, ribosomal structure and biogenesis]
200	RplO	J	Ribosomal protein L15 [Translation, ribosomal structure and biogenesis]
201	SecY	U	Preprotein translocase subunit SecY [Intracellular trafficking and secretion]
203	RplQ	J	Ribosomal protein L17 [Translation, ribosomal structure and biogenesis]
211	RpmA	J	Ribosomal protein L27 [Translation, ribosomal structure and biogenesis]
216	PrfA	J	Protein chain release factor A [Translation, ribosomal structure and biogenesis]
222	RplL	J	Ribosomal protein L7/L12 [Translation, ribosomal structure and biogenesis]
228	RpsP	J	Ribosomal protein S16 [Translation, ribosomal structure and biogenesis]
238	RpsR	J	Ribosomal protein S18 [Translation, ribosomal structure and biogenesis]
244	RplJ	J	Ribosomal protein L10 [Translation, ribosomal structure and biogenesis]
256	RplR	J	Ribosomal protein L18 [Translation, ribosomal structure and biogenesis]
268	RpsT	J	Ribosomal protein S20 [Translation, ribosomal structure and biogenesis]
275	MraW	M	Predicted S-adenosylmethionine-dependent methyltransferase involved in cell envelope biogenesis
285	FolC	H	Folylpolyglutamate synthase [Coenzyme metabolism]
292	RplT	J	Ribosomal protein L20 [Translation, ribosomal structure and biogenesis]
313	YraL	R	Predicted methyltransferases [General function prediction only]
319	YbeY	R	Predicted metal-dependent hydrolase [General function prediction only]
335	RplS	J	Ribosomal protein L19 [Translation, ribosomal structure and biogenesis]
336	TrmD	J	tRNA-(guanine-N1)-methyltransferase [Translation, ribosomal structure and biogenesis]
354	YgfZ	R	Predicted aminomethyltransferase related to GcvT [General function prediction only]
359	RplI	J	Ribosomal protein L9 [Translation, ribosomal structure and biogenesis]
360	RpsF	J	Ribosomal protein S6 [Translation, ribosomal structure and biogenesis]
361	InfA	J	Translation initiation factor 1 (IF-1) [Translation, ribosomal structure and biogenesis]
445	GidA	D	NAD/FAD-utilizing enzyme apparently involved in cell division [Cell division and chromosome partitioning
481	LepA	M	Membrane GTPase LepA [Cell envelope biogenesis, outer membrane]
482	TrmU	J	Predicted tRNA(5-methylaminomethyl-2-thiouridylate) methyltransferase [Translation, ribosomal struct.]
486	ThdF	R	Predicted GTPase [General function prediction only]
495	LeuS	J	Leucyl-tRNA synthetase [Translation, ribosomal structure and biogenesis]

504	PyrG	F	CTP synthase (UTP-ammonia lyase) [Nucleotide transport and metabolism]
522	RpsD	J	Ribosomal protein S4 and related proteins [Translation, ribosomal structure and biogenesis]
525	ValS	J	Valyl-tRNA synthetase [Translation, ribosomal structure and biogenesis]
532	InfB	J	Translation initiation factor 2 (IF-2; GTPase) [Translation, ribosomal structure and biogenesis]
533	QRI7	O	Metal-dependent proteases with possible chaperone activity [Posttranslational modification, chaperones]
536	Obg	R	Predicted GTPase [General function prediction only]
539	RpsA	J	Ribosomal protein S1 [Translation, ribosomal structure and biogenesis]
571	Rnc	K	dsRNA-specific ribonuclease [Transcription]
592	DnaN	L	DNA polymerase sliding clamp subunit (PCNA homolog) [DNA replication, recombination, and repair]
691	SmpB	O	tmRNA-binding protein [Posttranslational modification, protein turnover, chaperones]
707	MurG	M	UDP-N-acetylglucosamine:LPS N-acetylglucosamine transferase [Cell envelope biogenesis]
751	GlyS	J	Glycyl-tRNA synthetase, beta subunit [Translation, ribosomal structure and biogenesis]
771	MurD	M	UDP-N-acetylmuramoylalanine-D-glutamate ligase [Cell envelope biogenesis, outer membrane]
806	RimM	J	RimM protein, required for 16S rRNA processing [Translation, ribosomal structure and biogenesis]
812	MurB	M	UDP-N-acetylmuramate dehydrogenase [Cell envelope biogenesis, outer membrane]
816	YqgF	L	Predicted endonuclease involved in recombination (possible Holliday junction resolvase) [DNA recomb.]
849	FtsA	D	Actin-like ATPase involved in cell division [Cell division and chromosome partitioning]
858	RbfA	J	Ribosome-binding factor A [Translation, ribosomal structure and biogenesis]
1185	Pnp	J	Polyribonucleotide nucleotidyltransferase (polynucleotide phosphorylase) [Translation, ribosomal]
1207	GlmU	M	N-acetylglucosamine-1-phosphate uridyltransferase [Cell envelope biogenesis, outer membrane]
1214	YeaZ	O	Inactive homolog of metal-dependent proteases, putative molecular chaperone [Posttrans./chaperones]
1825	RplY	J	Ribosomal protein L25 (general stress protein Ctc) [Translation, ribosomal structure and biogenesis]
1841	RpmD	J	Ribosomal protein L30/L7E [Translation, ribosomal structure and biogenesis]
1862	YajC	U	Preprotein translocase subunit YajC [Intracellular trafficking and secretion]
1932	SerC	HE	Phosphoserine aminotransferase [Coenzyme metabolism / Amino acid transport and metabolism]
1949	Orn	A	Oligoribonuclease (3'->5' exoribonuclease) [RNA processing and modification]
2924	YggX	S	Uncharacterized protein conserved in bacteria [Function unknown]
