# Supplementary material for: Comparing Patterns of Natural Selection across Species Using Selective Signatures
Source: PLoS Genet. 2008 Feb 8;4(2):e23. doi: 10.1371/journal.pgen.0040023 (PMC2233676; doi:10.1371/journal.pgen.0040023)
Supplement: Supplementary file 4 [file pgen.0040023.st002.pdf]

Table S2a. Taxonomy IDs of species used in this study.

Taxonomy ID	Species Name
107806	*Buchnera aphidicola str. APS*
224915	*Buchnera aphidicola str. Bp*
198804	*Buchnera aphidicola str. Sg*
203907	*Candidatus Blochmannia floridanus*
167879	*Colwellia psychrerythraea 34h*
227377	*Coxiella burnetii RSA*
218491	*Erwinia carotovora SCRI1043*
83333	*Escherichia coli K12*
233412	*Haemophilus ducreyi35000HP*
71421	*Haemophilus influenzae Rd KW20*
283942	*Idiomarina loihiensis L2TR*
221988	*Mannheimia succiniciproducens MBEL55E*
747	*Pasteurella multocida*
298386	*Photobacterium profundum SS9*
243265	*Photorhabdus luminescens TTO1*
208964	*Pseudomonas aeruginosa PAO1*
220664	*Pseudomonas fluorescens Pf-5*
160488	*Pseudomonas putida KT2440*
223283	*Pseudomonas syringae*
99287	*Salmonella typhimurium LT2*
211586	*Shewanella oneidensis MR-1*
666	*Vibrio cholerae*
312309	*Vibrio fischeri ES114*
223926	*Vibrio parahaemolyticus RIMD2210633*
216895	*Vibrio vulnificus CMCP6*
36870	*Wigglesworthia glossinidia*
190485	*Xanthomonas campestris ATCC33913*
291331	*Xanthomonas oryzae pv. oryzae KACC103*
160492	*Xylella fastidiosa 9a5c*
273123	*Yersinia pestis IP32953*

Table S2b. Taxonomy IDs of strains used in McDonald-Kreitman tests.

Taxonomy ID	Species Name
358709	*Escherichia coli 101-1*
362663	*Escherichia coli 536*
405955	*Escherichia coli APEC O1*
37762	*Escherichia coli B*
344601	*Escherichia coli B171*
340184	*Escherichia coli B7A*
199310	*Escherichia coli CFT073*
340186	*Escherichia coli E110019*
340185	*Escherichia coli E22*
331111	*Escherichia coli E24377A*
340197	*Escherichia coli F11*
331112	*Escherichia coli HS*
83333	*Escherichia coli K12*
155864	*Escherichia coli O157:H7 EDL933*
386585	*Escherichia coli O157:H7 str. Sakai*
364106	*Escherichia coli UTI89*
316407	*Escherichia coli W3110*
344609	*Shigella boydii BS512*
300268	*Shigella boydii Sb227*
300267	*Shigella dysenteriae Sd197*
198215	*Shigella flexneri 2a str. 2457T*
198214	*Shigella flexneri 2a str. 301*
373384	*Shigella flexneri 5 str. 8401*
300269	*Shigella sonnei Ss046*
	**outgroup:**
99287	*Salmonella typhimurium LT2*
