# Supplementary material for: Comparing Patterns of Natural Selection across Species Using Selective Signatures
Source: PLoS Genet. 2008 Feb 8;4(2):e23. doi: 10.1371/journal.pgen.0040023 (PMC2233676; doi:10.1371/journal.pgen.0040023)
Supplement: Supplementary file 5 [file pgen.0040023.st003.pdf]

Table S3a. Enrichment of COG functional categories in top 10% fast- or slow-evolving sets of genes

Species	COG function	Enrichment	Bonferonni-corrected P-value
Haemophilus ducreyi	Ribosome & Translation	slow	<0.0001
C. Blochmannia floridanus	Ribosome & Translation	fast	<0.0001
Xylella fastidiosa	Ribosome & Translation	fast	<0.0001
Escherichia coli K12	Motlity & Secretion	fast	<0.001
Pasteurella multocida	Ribosome & Translation	slow	<0.001
Photorhabdus luminescens	Motlity & Secretion	fast	<0.001
Mannheimia succiniciproducens	Ribosome & Translation	slow	<0.001
Photorhabdus luminescens	Ribosome & Translation	slow	<0.001
Vibrio parahaemolyticus	Amino acid metabolism	slow	<0.01
Haemophilus influenzae	Ribosome & Translation	slow	<0.01
Buchnera aphidicola str. APS	Ion transport & metabolism	slow	<0.01
Wigglesworthia glossinidia	Coenzyme metabolism	slow	<0.01
Haemophilus ducreyi	Cell Division	fast	<0.05
Vibrio vulnificus	Nucleic acid metabolism	slow	<0.05
Yersinia pestis	Motlity & Secretion	fast	<0.05
Idiomarina loihiensis	Carbohydrate metabolism	fast	<0.05
Xylella fastidiosa	Energy production	slow	<0.05
Idiomarina loihiensis	Amino acid metabolism	fast	<0.05
Photobacterium profundum	Cell Division	fast	<0.05

Table S3a Legend:
Fast and slow-evolving sets of genes were defined as those with the highest or lowest 10% values of v in a genome, with a Z-score threshold of 1.0. A hypergeometric test was performed to test whether each functional category was enriched in the fast or slow set, relative to the expected fraction of that category in the whole genome. P-values are Boneronni-corrected for tests on 16 COG categories.

Table S3b. List of fast-evolving flagellar genes in 3 species of Enterobacteria

COG	Name	*E. coli K12*	*P. luminescens*	*Y. pestis*	Description
1377	FlhB	*	*		Flagellar biosynthesis pathway
1684	FliR	*	*		Flagellar biosynthesis pathway
3418	FlgN	*	*	*	Flagellar biosynthesis/type III secretory pathway chaperone
4787	FlgF	*	*		Flagellar basal body rod protein
1261	FlgA	*	*		Flagellar basal body P-ring biosynthesis protein
3190	FliO	*			Flagellar biosynthesis protein
4967	PilV	*		*	Tfp pilus assembly protein
1815	FlgB	*			Flagellar basal body protein
1345	FliD	*			Flagellar capping protein
1516	FliS	*	*		Flagellin-specific chaperone
4786	FlgG		*		Flagellar basal body rod protein
1706	FlgI		*		Flagellar basal body P-ring protein
1677	FliE		*		Flagellar hook basal body protein
2805	PilT		*	*	Tfp pilus assembly protein, pilus retraction ATPase
4969	PilA			*	Tfp pilus assembly protein, major pilin
1989	PulO			*	Type II secretory pathway, prepilin signal peptidase PulO and related peptidases

Table S3b Legend:
The COG function "Motility & Secretion" is enriched in the fast-evolving set of genes in 3 species of Enterobacteria: *Escherichia coli K12, Photorhabdus luminescens*, and *Yersinia pestis*.
The genes responsible for this trend are all involved in flagellar functions. Different genes are fast-evolving in each species: these are denoted by asterices in the relevant column.
