# Supplementary material for: Comparing Patterns of Natural Selection across Species Using Selective Signatures
Source: PLoS Genet. 2008 Feb 8;4(2):e23. doi: 10.1371/journal.pgen.0040023 (PMC2233676; doi:10.1371/journal.pgen.0040023)
Supplement: Supplementary file 6 [file pgen.0040023.st004.pdf]

Supplementary Table 4. Evidence for site-specific changes in dN/dS in *Idiomarina* genes.

			Test A: Selection on *Idiomarina*		Test B: Site-specific selection on *Idiomarina*	
**Pathway**	**Gene**	**COG**	**Different dN/dS in *Idiomarina* ?**	**dN/dS in *Idiomarina* (relative to other lineages)**	**Sites with higher dN/dS in *Idiomarina*?**	**Proportion of sites with higher dN/dS in *Idiomarina* (dN/dS > 1 in bold)**
**Glycolysis**	*Pgi*	166	yes*	0.0066 (lower)	yes***	0.40
	Fbp	158	yes**	0.0022 (lower)	yes***	0.18
	Eno	148	yes***	0.0017 (lower)	yes***	**0.08**
	TpiA	149	yes*	0.0056 (lower)	yes***	**0.26**
	Pfk	205	yes*	0.0038 (lower)	yes***	0.30
	NagE	2190	no	0.0185	yes***	0.75
**Phenylalanine biosynthesis**	*AroQ*	757	yes*	0.0028 (lower)	yes*	0.07
	AroB	337	no	0.0044	yes***	0.23
	AroE	169	no	0.0055	yes***	**0.16**
	PheA	77	no	0.0115	yes**	0.45

Likelihood ratio test: * $P < 0.05$, ** $P < 0.005$, *** $P < 0.00001$

To support the high values of ν observed in glycolysis and phenylalanine biosynthesis in *Idiomarina*, we analyzed these genes in PAML under 5 different evolutionary models: (1) No branch variation (single value of dN/dS for the whole tree); no site variation, (2) Different dN/dS allowed in the *Idiomarina* branch (two values of dN/dS in the tree); no site variation, (3) No branch variation; variable selective pressure among sites allowed (3 categories of sites, each allowed a different dN/dS), (4) Different dN/dS allowed in *Idiomarina*; variable selective pressure among sites allowed (3 categories of sites for *Idiomarina* and 3 for the rest of the tree, each allowed a different dN/dS), and (5) Same as Model 4, but fixing dN/dS not to exceed 1. In Test A, Model 2 is compared with Model 1 in a likelihood ratio test (compare $2(L_2 - L_1)$ to χ^2 distribution with degrees of freedom = # parameters in Model 2 – Model 1). In effect, this tests whether *Idiomarina* has a different dN/dS, averaged over all sites in the gene, than other lineages. It should be noted that for all genes in the table above, dS is saturated (> 50 substitutions per site), perhaps explaining the relatively low average values of dN/dS. In Test B, Model 4 is compared to Model 3 in a likelihood ratio test (compare $2(L_4 - L_3)$ to χ^2 distribution with degrees of freedom = # parameters in Model 4 – Model 3). For all 10 genes, this test supports sites under different selection in *Idiomarina*. In all cases, there is at least on site category with higher dN/dS in *Idiomarina* than other lineages. We performed a final test to determine whether there were some sites in *Idiomarina* with dN/dS > 1. In this test, we compared Model 4 to Model 5 in a likelihood ratio test (compare $2(L_4 - L_5)$ to χ^2 distribution with degrees of freedom = # parameters in Model 4 – Model 5). For genes with significant evidence ($P <$ 0.05) for sites with dN/dS > 1, the proportion of sites with elevated dN/dS in *Idiomarina* (far right column) is shown in bold.
