# Supplementary material for: Comparing Patterns of Natural Selection across Species Using Selective Signatures
Source: PLoS Genet. 2008 Feb 8;4(2):e23. doi: 10.1371/journal.pgen.0040023 (PMC2233676; doi:10.1371/journal.pgen.0040023)
Supplement: Supplementary file 7 [file pgen.0040023.st005.pdf]

Supplementary Table 5. Genes predicted under selection in *E. coli* by selective signatures and the MK test

COG	$\log_2 \nu$		$\log_2$ FI		dN/dS	Func	Description
112	2.13	**	2.47	*	0.12	E	serine hydroxymethyltransferase
118	2.02	**	0.82		0.07	E	imidazole glycerol phosphate synthase subunit HisH
150	1.96	**	0.69		0.03	F	phosphoribosylaminoimidazole synthetase
2973	1.86	*	0.41		0.00	K	Trp operon repressor
157	1.86	**	0.68		0.02	H	nicotinate-nucleotide pyrophosphorylase
2980	1.73	**	0.43		0.07	M	minor lipoprotein
704	1.54	*	0.84		3.21	P	negative regulator of PhoR/PhoB two-component regulator
357	1.51	**	0.61		0.02	M	glucose-inhibited division protein B
99	1.48	*	0.74		0.24	J	30S ribosomal protein S13
649	1.48	*	1.87	*	0.02	C	NADH:ubiquinone oxidoreductase, chain C,D
196	1.33	**	0.54		0.03	H	hypothetical protein
414	1.30	*	2.02	*	0.04	H	pantoate--beta-alanine ligase
2109	1.28	*	0.50		0.00	H	cob(I)yrinic acid a,c-diamide adenosyltransferase
3123	1.28	*	0.94		0.02	S	hypothetical protein
1214	1.27	**	0.94		0.05	O	predicted peptidase
3159	1.21	*	0.90		0.09	S	hypothetical protein
850	1.19	**	0.37		0.06	D	septum formation inhibitor
221	1.16		1.03		0.09	C	inorganic pyrophosphatase
2861	1.15	*	0.85		0.63	S	orf, hypothetical protein
1983	1.12	*	0.93		0.06	KT	DNA-binding transcriptional activator
529	1.11		0.55		0.05	P	adenylylsulfate kinase
149	1.07	*	2.08		0.08	G	triosephosphate isomerase
205	1.05	*	0.89		0.03	G	6-phosphofructokinase
3076	1.03		0.30		0.02	S	hypothetical protein
229	0.97	*	0.67		0.02	O	methionine sulfoxide reductase B
540	0.96	*	1.68		0.02	F	aspartate carbamoyltransferase catalytic subunit
1005	0.95		1.82	*	0.16	C	NADH dehydrogenase subunit H
751	0.88	*	2.09	*	0.05	J	glycyl-tRNA synthetase subunit beta
17	0.86		1.00		0.05	J	asparaginyl-tRNA synthetase
481	0.85		0.94		0.02	M	GTP-binding protein LepA
1666	0.84		2.52	*	0.07	S	orf, hypothetical protein
344	0.82	*	1.74		0.08	S	hypothetical protein
682	0.80	*	1.93	*	2.50	M	prolipoprotein diacylglyceryl transferase
2377	0.80	**	0.37		0.01	O	anhydro-N-acetylmuramic acid kinase
554	0.74		0.27		0.03	C	glycerol kinase
151	0.73		0.29		4.33	F	phosphoribosylamine--glycine ligase
233	0.73		0.95		0.07	J	ribosome releasing factor
249	0.69	*	1.51	*	0.02	L	DNA mismatch repair protein
66	0.69		0.32		0.08	E	isopropylmalate isomerase small subunit
212	0.68	*	0.61		0.01	H	putative ligase
1207	0.64	*	0.91		0.06	M	bifunctional N-acetylglucosamine-1-phosphate uridyltransferase/glucosamine-1-phosphate acetyltransferase
2908	0.64	*	0.44		0.06	S	UDP-2,3-diacylglucosamine hydrolase
165	0.63		0.37		0.09	E	argininosuccinate lyase
468	0.63		0.67		0.01	L	recombinase A
3004	0.62		0.66		0.00	P	pH-dependent sodium/proton antiporter
2360	0.62	*	0.56		0.04	O	leucyl/phenylalanyl-tRNA--protein transferase
1485	0.60	*	0.37		0.04	R	conserved protein with nucleoside triphosphate hydrolase domain
106	0.58		0.32		0.02	E	N-(5'-phospho-L-ribosyl-formimino)-5-amino-1-(5'- phosphoribosyl)-4-imidazolecarboxamide isomerase
323	0.58	*	0.60		0.02	L	DNA mismatch repair protein
418	0.58	*	0.30		0.02	F	dihydroorotase
629	0.56		1.41		0.06	L	single-strand DNA-binding protein

2968	0.52		1.34		0.02	S	hypothetical protein
519	-0.51	*	-1.10		0.01	F	bifunctional GMP synthase/glutamine amidotransferase protein
2917	-0.52		-1.13		0.01	D	intracellular septation protein A
2925	-0.52	*	-1.45	*	0.01	L	exonuclease I
262	-0.57	*	-0.91		0.77	H	dihydrofolate reductase
1188	-0.59		-1.46		2.06	J	ribosome-associated heat shock protein Hsp15
275	-0.65	*	-0.98		0.01	M	S-adenosyl-methyltransferase
1314	-0.71	*	-0.87		0.09	U	protein-export membrane protein
4121	-0.73	*	-0.76	*	0.08	S	putative peptidase
324	-0.74		-1.90	*	0.05	J	tRNA delta(2)-isopentenylpyrophosphate transferase
2976	-0.76	*	-1.58		0.04	S	hypothetical protein
93	-0.81		-2.32		0.15	J	50S ribosomal protein L14
1212	-0.83	*	-1.61	*	0.00	M	3-deoxy-manno-octulosonate cytidylyltransferase
777	-0.99	*	-2.35	*	0.05	I	acetyl-CoA carboxylase subunit beta
774	-1.04	*	-1.26		0.04	M	UDP-3-O-[3-hydroxymyristoyl] N-acetylglucosamine deacetylase
5633	-1.05	*	-2.65	*	0.08	R	hypothetical protein
2190	-1.11	*	-0.99		0.03	G	glucose-specific PTS system enzyme IIA component
4657	-1.13	*	-2.93	*	0.03	C	Na(+)-translocating NADH-quinone reductase subunit E
360	-1.15	*	-3.00	*	0.06	J	30S ribosomal protein S6
563	-1.25	*	-0.85		0.10	F	adenylate kinase
466	-1.28	*	-3.55	*	0.01	O	DNA-binding ATP-dependent protease La
3151	-1.37	*	-2.46		0.02	S	predicted dehydrogenase
4785	-1.37	**	-0.99		0.08	R	hypothetical protein
101	-1.41	*	-1.14		1.40	J	tRNA pseudouridine synthase A
821	-1.50	*	-0.81		0.01	I	4-hydroxy-3-methylbut-2-en-1-yl diphosphate synthase
2983	-1.52	*	-1.84	*	0.00	S	orf, hypothetical protein
509	-1.58	*	-2.62	*	0.01	E	glycine cleavage system protein H
287	-1.58	*	-0.88		1.62	E	fused chorismate mutase T/prephenate dehydrogenase
788	-1.63	*	-1.34		1.28	F	formyltetrahydrofolate deformylase
634	-1.69	*	-1.68		0.02	F	hypoxanthine phosphoribosyltransferase
283	-1.69	*	-1.75		0.01	F	cytidylate kinase
131	-1.75	*	-1.71	*	0.02	E	imidazoleglycerolphosphate dehydratase and histidinol-phosphate phosphatase
452	-2.01	*	-1.05		0.03	H	flavoprotein affecting synthesis of DNA and pantothenate metabolism
41	-2.43	*	-1.23		0.04	F	phosphoribosylaminoimidazole carboxylase catalytic subunit
3317	-2.51	*	-1.52	*	0.03	M	lipoprotein-34
575	-2.60	*	-1.77	*	1.38	I	CDP-diglyceride synthetase
779	-2.62	*	-1.15		0.03	S	orf, hypothetical protein
539	-2.71	*	-1.32		0.02	J	30S ribosomal protein S1
3117	-2.77	*	-1.72	*	0.00	S	hypothetical protein
103	-2.78	*	-1.42		0.06	J	30S ribosomal protein S9
55	-2.86	*	-1.16		0.01	C	F0F1 ATP synthase subunit beta
1385	-2.89	*	-1.68	*	1.34	S	orf, hypothetical protein

Legend. Genes are listed only when the MK test and selective signatures agree, using the same criteria as Figure 7 in the main text ($\log_2$FI > 0.26 and $\log_2\nu > 0.5$, or $\log_2$FI < -0.74 and $\log_2\nu < -0.5$). For ν, * denotes Z score > 1, ** Z > 2. For FI, * denotes $\chi^2 > 3.84$ ($P < 0.05$ with 1 d.f.).

COG functional category key:

C	Energy Production	K	Transcription
D	Cell Division	L	DNA replication, repair & modification
E	Amino Acid Metabolism	M	Cell Envelope
F	Nucleic Acid Metabolism	N	Motility & Secretion
G	Carbohydrate Metabolism	O	Protein Modification & degradation
H	Coenzyme Metabolism	P	Ion Transport & Metabolism
I	Lipid Metabolism	Q	Secondary Metabolism
J	Ribosome & Translation	R	General Function
S	Unknown	T	Signal Transduction
